# Phylogenomics of the *Maverick* Virus-Like Mobile Genetic Elements of Vertebrates

**DOI:** 10.1093/molbev/msaa291

**Published:** 2021-01-22

**Authors:** Jose Gabriel Nino Barreat, Aris Katzourakis

**Affiliations:** Department of Zoology, University of Oxford, Oxford, United Kingdom

**Keywords:** *Mavericks*, mobile genetic elements, dsDNA viruses, paleovirology, vertebrates

## Abstract

*Mavericks* are virus-like mobile genetic elements found in the genomes of eukaryotes. Although *Mavericks* encode capsid morphogenesis homologs, their viral particles have not been observed. Here, we provide new evidence supporting the viral nature of *Mavericks* and the potential existence of virions. To this end, we conducted a phylogenomic analysis of *Mavericks* in hundreds of vertebrate genomes, discovering 134 elements with an intact coding capacity in 17 host species. We reveal an extensive genomic fossil record in 143 species and date three groups of elements to the Late Cretaceous. Bayesian phylogenetic analysis using genomic fossil orthologs suggests that *Mavericks* have infected osteichthyans for ∼419 My. They have undergone frequent cross-species transmissions in cyprinid fish and all core genes are subject to strong purifying selection. We conclude that vertebrate *Mavericks* form an ancient lineage of aquatic dsDNA viruses which are probably still functional in some vertebrate lineages.

## Introduction

*Mavericks* are mobile genetic elements found integrated in the genomes of most eukaryotes, with the notable exception of mammals and land plants (Kapitonov and Jurka 2006; Pritham et al. 2007). They were identified initially from the presence of a distinct integrase that was thought to be of cellular origin (the “c-integrases”) (Gao and Voytas 2005). However, closer inspection of the surrounding genomic architecture revealed the existence of additional open reading frames and flanking inverted repeats, a telltale sign of DNA transposons (Feschotte and Pritham 2005). Nevertheless, there is mounting evidence based on sequence and structural comparisons, that *Mavericks* share striking features and a close evolutionary relationship with viruses.

Indeed, phylogenetic and comparative genomic analyses have placed *Mavericks* in the PRD1–adenovirus lineage, a diverse assemblage of mobile genetic elements that infect bacteria, archaea, and eukaryotes (Krupovic and Bamford 2008). *Mavericks* encode a family B protein-primed DNA polymerase, an adenoviral-like protease, retroviral-like integrase, and DNA packaging ATPase (Pritham et al. 2007). In addition, two conserved genes were found to encode proteins homologous to the double and single jelly-roll capsid proteins of diverse dsDNA viruses (Krupovic et al. 2014). The evolutionary history of the PRD1–adenovirus lineage may represent the most remarkable diversification ever seen in the virosphere, both in terms of ecology and genome complexity. Both capsid-encoding and capsid-less elements are found in this group, which comprises bacteriophages (tectiviruses, corticoviruses), archaeoviruses (turriviruses), the nucleocytoplasmatic large DNA viruses (NCLDVs, including the giant viruses), virophages, mitochondrial and cytoplasmic linear plasmids, adenoviruses, *Tlr*-elements, *Polinton*-like viruses, and *Mavericks* (Koonin and Krupovic 2017).

Although viral particles for *Mavericks* have not been observed, the fact that they encode the full repertoire of genes required for capsid morphogenesis suggests they are viruses (Krupovic et al. 2014). This is consistent with a scenario where these elements have retained the ancestral capsid-encoding capacity of the PRD1–adenovirus lineage, whereas the capsid-less forms have originated on several occasions via reductive evolution. Precisely, the defining feature of viruses is the ability to form a protective protein shell used in the horizontal transfer of parasitic genetic replicons. It is this feature which sets them apart from other mobile genetic elements such as viroids, transposons, and plasmids. Therefore, pinpointing the precise nature of *Mavericks*, whether they are viruses or transposons, is a fundamental question with important implications in understanding the evolutionary biology of the PRD1–adenovirus lineage.

The current classification of *Mavericks* groups them into two clades, group-I and group-II, which are paraphyletic (Krupovic and Koonin 2015). There is indication that horizontal transfers have occurred during their evolutionary history, for example, both group-I and group-II elements coexist in the genomes of *Nasonia vitripennis* (Hymenoptera), *Tribolium castaneum* (Coleoptera), and *Nematostella vectensis* (Cnidaria) (Haapa-Paananen et al. 2014). Similarly, the *Mavericks* in two species of drosophilid flies, *Drosophila bipectinata* and *D. eugracilis*, were shown to descend from different lineages (Haapa-Paananen et al. 2014). In vertebrates, the elements that have been analyzed seem to have been inherited vertically within their hosts (Haapa-Paananen et al. 2014). As such, it seems that *Mavericks* may undergo evolutionary dynamics similar to retroviruses, combining vertical inheritance in the host-germline with cross-species transmissions. Given that hundreds of vertebrate assemblies are now available for genome mining, vertebrates represent a suitable group of animals to test these ideas on the evolutionary biology of *Mavericks*.

Here, we investigate the viral nature of vertebrate *Mavericks* by an extensive, in depth phylogenomic analysis. This approach allows us to gain insights into the genetic diversity, abundance, taxonomic distribution, and the evolutionary history of *Mavericks* in vertebrates.

## Results

### *Mavericks* Occur in All Major Osteichthyian Lineages Except for Mammals

We found evidence of *Mavericks* in the genomes of ray-finned fish (Actinopterygii), coelacanths (Coelacanthi), amphibians (Lissamphibia), lepidosaurs (Lepidosauria), turtles (Testudines), crocodiles (Crocodilia), and birds (Aves). No hits mapped to the genomes of mammals, except for the unplaced genomic scaffold NW_019367942.1 in the cat (*Felis catus*) assembly, which appears to be a contaminant from fish. This sequence shows consistent similarity to fish *Mavericks* in Censor (Jurka et al. 1996) and contains a Harbinger DNA transposon which are not known in mammals (Kapitonov and Jurka 2004). Thus, we were able to map a total of 3,511 loci homologous to *Mavericks* in 143 species belonging to all major groups of Osteichthyes excluding mammals (supplementary excel file 1 and list 1, Supplementary Material online).

### The Vast Majority of Elements Are Mutationally Degraded

The majority of these loci (96%) represent sequences that have become eroded by host mutation and have lost their coding capacity. This could be seen in the Genewise annotations where stop codons, frameshift mutations, and large insertions (which Genewise detects as introns), were identified as well as in the lack of discernible TIRs in 2,166 elements. We did find a total of 134 elements in 17 species which encode eight conserved and intronless ORFs (or seven in *Xenopus tropicalis*), terminal inverted repeats, and 6-bp target site duplications. Therefore, the latter seem to be potentially active *Mavericks*, which we call “intact,” whereas the former are probably genomic fossils.

### The Genomic Architecture of Vertebrate *Mavericks* Is Conserved

**Fig. 1.**
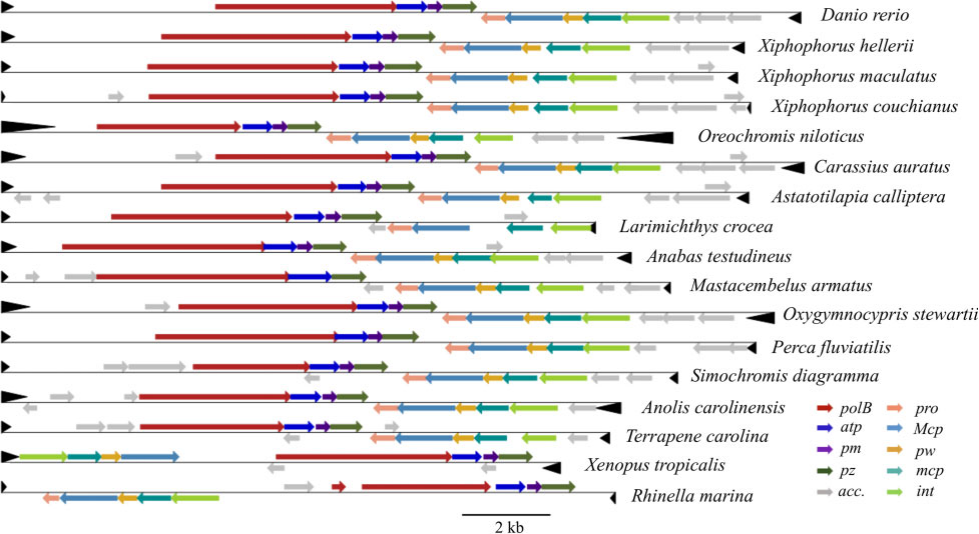
The genomic architectures of vertebrate *Mavericks* are conserved. Elements from teleosts are shown above and those from tetrapods below. Colored arrows indicate open reading frames, black arrowheads indicate TIRs. See text for details. *ppolb*, protein-primed DNA polymerase; *atp*, ATPase; *pm*, PM; *pz*, PZ; *pro*, protease; *Mcp*, major capsid protein; *pw*, PW; *mcp*, minor capsid protein; *int*, integrase; acc., accessory genes. Scale bar = 2,000 bp.

The genomic architectures of the intact *Mavericks* of vertebrates are generally conserved (fig. 1). The genes are arranged in two modules: a first module containing the genes coding for the protein-primed DNA polymerase, ATPase, and the PZ protein, and a second module coding for the integrase, minor capsid protein, the PW protein, major capsid protein, and protease. We discovered an additional conserved ORF of uncertain function which lies between *atp* and *pz*: *pm*. The two gene modules occupy different strands and both are oriented toward the center of the genome. Most elements have two accessory regions flanking the core modules where more ORFs encoding diverse proteins can be found. Interestingly, the modules in the elements of the cane toad *Rhinella marina* point away from each other, whereas in the western clawed frog *X. tropicalis*, they occupy the same strand; accessory regions appear to be absent in these *Mavericks*. Sizes of the intact elements ranged from 12,723 to 24,620 bp with a mean and SD of 16,862 ± 1,678 bp; the sizes of TIRs also varied, they comprised between 46 and 1,447 bp with a mean and SD of 402 ± 276 bp (supplementary fig. S1, Supplementary Material online).

The genomes of vertebrate *Mavericks* have conserved 6-bp end-motifs at the termini. The most common motif is an AGT trinucleotide repeated twice at the 5′-end which occurs together with an ACT trinucleotide repeated twice at the 3′-end; or 5′-(AGT)_2_//(ACT)_2_-3′, where “//” is the *Maverick* sequence internal to the termini. Another common motif is an AG dinucleotide repeated three times with a downstream CT repeat, 5′-(AG)_3_//(CT)_3_-3′. A new motif type was found in *Mavericks* of the Asian swamp eel, *Monopterus albus*, which consists of dinucleotides followed by a single conserved base of the form 5′-(AC)_3_A//T(GT)_3_-3′. In all types, these conserved positions are characterized by an information content >1 bit which decreases markedly as one moves away from the motifs into the DNA of the host, and to a lesser degree inward to the internal sequence (the bit content of a site is calculated from the formula: information content = 2 $+\;\sum p_{i}\log_{2}p_{i}$, where *p_i_* is the relative frequency of nucleotide *i* at that position; supplementary fig. S2, Supplementary Material online).

### *Mavericks* Mostly Attain Low Copy Numbers, but Have Amplified Enormously in a Few Genomes

**Fig. 2.**
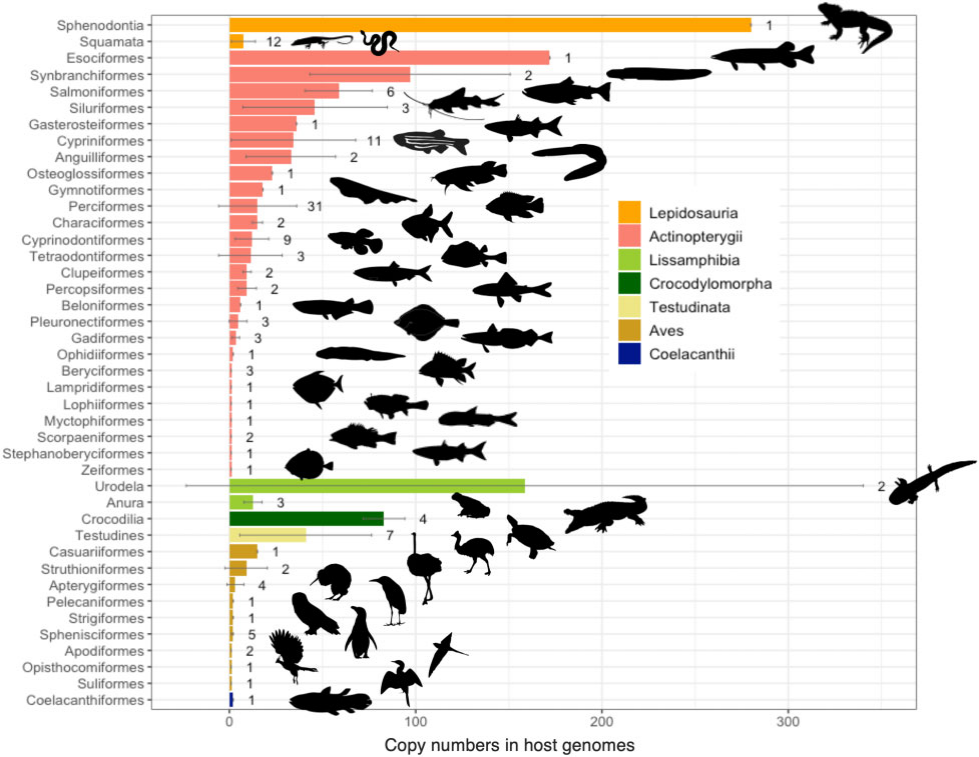
Copy numbers of *Mavericks* are low in most taxa. Major clades of vertebrates are color coded in the legend. Each bar shows the mean and SD, as well as the number of observations (analyzed genomes) in that Order, to the right. *N* = 143. Silhouettes obtained from PhyloPic.

In general, *Mavericks* attain low copy numbers in the genomes of their vertebrate hosts, with an average of 22 and a median of eight elements (fig. 2). In some cases, they can be considerably more as in the genomes of the axolotl *Ambystoma mexicanum* (=287), the tuatara *Sphenodon punctatus* (=280), or the pike *Esox lucius* (=172). However, all the elements in these hosts seem to be defective since the genes for the protein-primed DNA polymerase are fragmented by stop codons/frameshift mutations. In other genomes both genomic fossils and intact elements can be found, which total 135 in the Asian swamp eel *Mastacembelus armatus* (11 intact copies), 123 in the Tibetan cyprinid *Oxygymnocypris stewartii* (38 intact copies), 105 in the Nile tilapia *Oreochromis niloticus* (5 intact copies), and 101 in the eastern box turtle *Terrapene carolina* (1 intact copy). Copy numbers of intact elements range from one in some genomes of cichlids, turtles, and amphibians to a maximum of 38 in *O. stewartii* (supplementary table S1, Supplementary Material online). Of a total of 134 elements, 129 were found in the genomes of teleosts, whereas only five were identified in tetrapods. Moreover, the intact *Mavericks* of teleosts are considerably more abundant (and variable) with a mean and SD of 9.92 ± 10.11 copies per genome, compared to 1.25 ± 0.50 copies per genome in tetrapods.

### Orthologous Loci Suggest Minimum Insertion Dates Spanning 3–95 Ma

Pairwise comparisons among all loci allowed us to identify 115 sequences in 54 groups of orthologs (supplementary excel file 2, Supplementary Material online). Orthologous loci were found in the genomes of crocodiles, paleognath birds, penguins, testudinoid turtles, perciform, anguilliform, cyprinid, and salmonid fish (supplementary fig. S3, Supplementary Material online). Some taxa have multiple ortholog groups, which indicate that independent integration events had already occurred in the genome of their most recent common ancestor. Three of our calibrations date back to the Late Cretaceous (∼80–95 Ma), whereas all others are from the Cenozoic Era (∼3–54 Ma). None of the ortholog groups contained intact *Maverick*s, they were all genomic fossils from defective elements. It is important to note that these ages reflect minimum conservative estimates of the age of the insertions, since they could have occurred at a point prior to the divergence of the host species (Katzourakis and Gifford 2010), although it is unlikely they are too far away in the past.

### *Mavericks* Have Infected Vertebrates for Hundreds of Millions of Years

The inferred time scale of evolution of vertebrate *Mavericks* was in the order of hundreds of millions of years. Estimated root ages vary between a mean of 454.73 and 231.86 Ma, with seven of the eight proteins suggesting a Palaeozoic origin for vertebrate *Mavericks*; the youngest estimate from the 95% probability densities (HPDs) was ∼177 Ma for the major capsid protein (Jurassic Period of the Mesozoic Era) and the oldest, ∼569 Ma for the PZ protein (Ediacaran Period of the Proterozoic Eon) (supplementary table S2, Supplementary Material online). Most evolutionary rates are in the order of ∼10^−9^ amino acid substitutions per site per year, with minimum rates in the order of ∼10^−10^ and the highest around ∼10^−7^ (supplementary table S2, Supplementary Material online). A limitation of our approach is that the fossil ortholog calibrations implicitly assume that *Mavericks* have been evolving mostly at the host neutral rate, since the branches after the calibration points largely represent host evolution. This would translate into an overestimation of the divergence times if the rates at which the sequences had been evolving were actually higher (e.g., in the case of exogenous ancestors). However, considering that *Mavericks* appear to persist mostly as endogenous copies in the genomes of their hosts, rates of this order of magnitude have been estimated using different approaches for other families of viruses (see Discussion) and that the deep topology of the pPOLB tree is consistent with several codivergence scenarios in the order of hundreds of millions of years (supplementary fig. S4, Supplementary Material online), it seems that our evolutionary rate and divergence time estimates are good approximations of reality.

### The Phylogeny of *Mavericks* Suggests Vertical Transmission as well as Horizontal Transfers

The tree from the pPOLB protein had the best overall support, with an average posterior probability of clades of 0.89. In this maximum clade credibility tree, the *Mavericks* from sarcopterygians and actinopterygians each form monophyletic groups (supplementary fig. S4, Supplementary Material online). *Mavericks* of tetrapods are the sister group to the coelacanths (posterior probability = 0.64), and the recently proposed Archelosauria was also recovered, that is, (Testudines, (Crocodilia, Aves)) with a posterior probability = 1. Similarly, we observe a sister relationship between Salmoniformes and Esociformes (pikes). These observations agree with the host topology and are consistent with the idea that some *Maverick* lineages have been inherited vertically throughout osteichthyan evolution; although we cannot rule out possible horizontal transfers with unsampled intermediates.

However, there are also apparent discordances with the host topology, most notably, the lepidosaur, perciform, and siluriform *Mavericks* are polyphyletic (supplementary fig. S4, Supplementary Material online), which also occurs for the elements in chelonians and the fish orders: Anguilliformes (true eels), Characiformes (piranhas and tetras), Pleuronectiformes (flatfish), Salmoniformes + Esociformes (two clades), Synbranchiformes (swamp eels), and Tetraodontiformes (ocean sunfish, pufferfish). This is also consistent with the paraphyly of *Mavericks* in the Cypriniformes (minnows and carps). In general, these patterns hold for the trees inferred independently from the other seven proteins, especially the monophyly of tetrapod *Mavericks* (see rooted trees in the Data Availability section). The most notable difference being that, in the other trees, the *Mavericks* of teleosts are paraphyletic to the ones in tetrapods and fall in two lineages, however, given that the posterior probability for the grouping of one of the teleost lineages as the sister to tetrapods is generally low (posterior probability = 0.51 ± 0.34), we cannot reject the monophyly of the *Mavericks* of teleosts. An independent phylogeny estimated exclusively from the pPOLB sequences of intact elements is in total agreement with these findings (supplementary fig. S5, Supplementary Material online), with a support >0.99 for all major clades, suggesting that our analysis is robust to the inclusion of degraded genomic fossils.

### *Mavericks* Have Switched Hosts Frequently in Cyprinid Fish

**Fig. 3.**
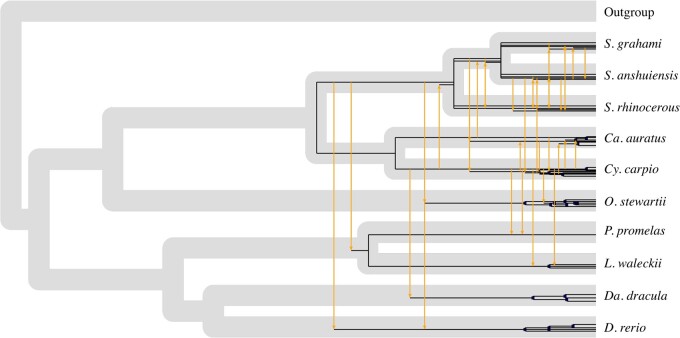
*Mavericks* have switched hosts frequently in cyprinid fish. The figure depicts the most parsimonious reconciliation of the cyprinid pPOLB tree onto the host phylogeny. Black lines represent vertical inheritance within the host genome and yellow arrows indicate host switch events. Genera abbreviations: *Sinocyclocheilus* (*S.*), *Carassius* (*Ca.*), *Cyprinus* (*Cy.*), *Oxygymnocypris* (*O.*), *Pimephales* (*P.*), *Leuciscus* (*L.*), *Danionella* (*Da.*), and *Danio* (*D.*).

We focused on cypriniform fish for the cophylogenetic and selection analyses, given that their *Mavericks* were not monophyletic, several species contained intact elements and high copy numbers were found in *O. stewartii*. The coevolutionary event with the highest probability inferred from the approximate Bayesian computation (ABC) analysis was cospeciation, with a mean probability of 0.64 ± 0.05, followed by host switching with a mean probability of 0.26 ± 0.06. Reconciliation of the cyprinid *Maverick* phylogeny with the host’s (using the transformed event probabilities as costs), suggests that although cospeciation and within-host amplification have been common evolutionary paths for the *Mavericks* of cyprinids, host switching has also occurred extensively (fig. 3). Moreover, species such as *Danionella dracula*, whose *Mavericks* are monophyletic, seem to all descend from an ancestral horizontal transfer, whereas in other host genomes multiple lineages coexist, some of which were inherited vertically and others by independent colonization events (e.g., *Danio rerio*, *O. stewartii*). We must recognize that these results rely on an accurate cyprinid host phylogeny; although we built a host tree consistent with several independent molecular works, these relationships have been problematic to resolve (Stout et al. 2016). However, we believe the presence of polyphyletic *Maverick* lineages in some genomes argues in favor of horizontal transfers even if the true host species phylogeny is unknown.

### The Core Genes of Cyprinid *Mavericks* Are under Strong Purifying Selection

As we have seen previously, although some *Mavericks* are intact most are degraded elements. Previous analyses had used consensus sequences and the selective forces affecting their evolution had not been explored (Kapitonov and Jurka 2006; Haapa-Paananen et al. 2014). Evidence of selection acting on the capsid morphogenesis homologs in a phylogenetic context, is a good indication that *Mavericks* have been an active group of endogenous viruses. Here, we found that the eight core genes of *Mavericks* in cyprinid fish are under strong purifying selection with *ω* ∼ 0.001 (table 1); meaning that for every 1,000 synonymous changes that are fixed, a single nonsynonymous change reaches fixation. The best models we fitted were either a single-ratio estimated *ω* (*int*, *Mcp*, *ppolb*, *pro*) or the two-ratio model with an *ω* for internal and terminal branches (*atp*, *mcp*, *pz*). The estimated *ω* for the internal branches was somewhat lower but in the same order of magnitude as the *ω* for terminal branches. Interestingly, *pw* was the single gene for which the two-ratio host switch model was favored.

**Table 1.** Maximum-Likelihood Estimates for the Nonsynonymous to Synonymous Ratios (*ω*) of the Eight Conserved Genes in Cyprinid *Mavericks.*

Gene	Best Model	*ω*	*P* Value
*atp*	2-ratios, *ω*_internal_/*ω*_terminal_	*ω*_internal_ = 0.00044 *ω*_terminal_ = 0.00066	0.000
*int*	1-ratio, *ω*-estimated	*ω* = 0.00138	0.000
*Mcp (py)*	1-ratio, *ω*-estimated	*ω* = 0.00181	0.000
*mcp (px)*	2-ratios, *ω*_internal_/*ω*_external_	*ω*_internal_ = 0.00074 *ω*_terminal_ = 0.00105	0.000
*ppolb*	1-ratio, *ω*-estimated	*ω* = 0.00099	0.000
*pro*	1-ratio, *ω*-estimated	*ω* = 0.00101	0.000
*pw*	2-ratios, *ω*_no-switch_/*ω*_switch_	*ω*_no-switch_ = 0.001255 *ω*_switch_ = 0.001494	0.000
*pz*	2-ratios, *ω*_internal_/*ω*_terminal_	*ω*_internal_ = 0.00102 *ω*_terminal_ = 0.00157	0.000

Note.—*P* values were derived from a χ^2^ comparison between the best and neutral models, a *P* value <0.05 indicates rejection of neutrality.

## Discussion

*Mavericks* are widespread across the genomes of osteichthyans but seem to have gone extinct in mammals. Their distribution is patchy and they mostly attain low copy numbers in most genomes. Given that *Mavericks* are large elements, it is possible that high copy numbers are selected against in host genomes as they pose a risk of deleterious ectopic recombination (Petrov et al. 2003). The hundreds of defective copies in the genomes of *A. mexicanum* and *S. punctatus* are perplexing, and it is still unclear which biological processes might be responsible for these amplification events or if they have exerted any impact on these organisms. The high copy numbers found in the axolotl could be related to the low deletion rates which have been detected in salamander genomes, which contribute to their large genome sizes as well (Sun et al. 2012).

Since few chondrichthyans were available for screening, the apparent absence of *Mavericks* from this group may reflect a sampling bias rather than an actual absence. A central theme that has emerged from our analysis is that most copies (96%) of vertebrate *Mavericks* are defective and thus, constitute a rich record of highly degraded genomic fossils. The observation that most intact elements were found in ray-finned fish is relevant since diverse families of giant viruses, virophages, and *Polinton*-like viruses (La Scola et al. 2008; Yutin et al. 2015; Yoshikawa et al. 2019), elements to which *Mavericks* are related, also infect aquatic organisms. Recently, Bellas and Sommaruga (2019) have shown that *Polinton*-like viruses are among the most abundant viruses from an alpine lake ecosystem in Austria. Haapa-Paananen et al. (2014) noticed that vertebrate *Mavericks* formed a clade with elements from cnidarians, echinoderms, and marine mollusks. Our comprehensive analysis across a broad diversity of vertebrates confirms the notion that water plays an important role in the transmission of *Mavericks*.

The genomic arrangement of core genes into two modules seems to be a universal characteristic of vertebrate *Mavericks*. No introns were observed in the genes of intact elements, which is in agreement with the work of Kapitonov and Jurka (2006). Although the *pz* and *pw* genes still have an uncharacterized function, their topological conservation indicates that they are required for the maintenance of *Mavericks* in the genomes of vertebrates. The *pm* gene we describe, is also conserved, implying a relevant role in the lifecycle of vertebrate *Mavericks*. Other conserved features of vertebrate *Mavericks* are the genome end-motifs and the sizes of intact elements. The end-motif repetitions allow the “jumping-back” mechanism required during protein-primed DNA replication in adenoviruses and tectiviruses (Caldentey et al. 1993; King and van der Vliet 1994), and would be expected to have the same function in *Mavericks* (Kapitonov and Jurka 2006). In *M. albus*, the existence of 14 defective copies with the unique AC-type indicates that the ancestral element of this motif was capable of replication. From the analysis of the element sizes, the variation around the mean size of about ∼17 kb is about 10%, suggesting a constrained genome size. In adenoviruses, departures from the wild-type genome size have been shown to affect the stability of the virus capsid (Smith et al. 2009).

We provide extensive evidence for the presence of *Mavericks* in the genomes of birds. Pritham et al. (2007) mentioned this possibility after finding sequence homology to the *Maverick* integrase in short genomic clones of *Apteryx australis*. Subsequently, Guizard et al. (2016) described remnants of *Mavericks* in two distinct loci of *Gallus gallus* with corresponding homologs in *Meleagris gallopavo*. In our analysis, we have found defective copies of *Mavericks* in the genomes of palaeognaths, penguins, swifts and hummingbirds, owls, cormorants, ibises, and in the hoatzin. As the *Mavericks* of birds form a well-supported monophyletic group and there are two loci in *Ap. australis* with seven of the eight core genes (one is lacking *pro*, the other *atp*), it seems likely that there were active *Mavericks* in the genome of the most recent common ancestor of extant birds. We also found diverse integrations in the genomes of nonavian reptiles which expand the known range of *Mavericks*, this is the case for the elements discovered in snakes (suborder Serpentes) and in turtles from the families Cheloniidae, Testudinidae, and Trionychidae, including an intact element from *T. carolina*.

Identification of orthologous endogenous viral elements from the Late Cretaceous is not unprecedented. Endogenous viral elements of comparable age have been described for bornaviruses in afrotherians (Katzourakis and Gifford 2010), an ERV-L retrovirus from placental mammals (Lee et al. 2013) and an hepadnavirus found in neoavian birds (Suh et al. 2013). From the Bayesian phylogenetic analyses, it is clear that these elements have infected vertebrates for hundreds of millions of years. In fact, we cannot rule out the presence of *Mavericks* in the genome of the most recent common ancestor of Osteichthyes, since the 95% highest probability densities for five proteins include the estimated age of the actinopterygian/sarcopterygian split at the Silurian/Devonian boundary, ∼419 Ma (Zhu et al. 2009). Similar time scales of virus evolution have been estimated for retroviruses under the power-law decay model (∼450 Ma) (Aiewsakun and Katzourakis 2017). It would appear, therefore, that the superlineage to which *Mavericks* belong is considerably older.

Analysis of mammalian and avian herpesvirus evolution has revealed substantial codivergence with their hosts, and under this assumption, their evolutionary rates have been estimated at 3 × 10^−9^ amino acid substitutions per site per year (McGeoch et al. 2000). By using the power-law model of evolutionary rates that has been used to study retrovirus macroevolution (Aiewsakun and Katzourakis 2017), and assuming that the sequences we analyzed diverged ∼419 Ma, we would expect to observe 2.426 × 10^−9^ amino acid substitutions per site per year. This expected value is remarkably similar to the mean rates we have estimated independently for all core proteins of *Mavericks*. These low estimates are consistent with the time-dependent rate phenomenon of molecular evolution: estimated evolutionary rates decrease as the divergence time between sequences increases, and this has also been shown to occur generally across viruses in different Baltimore groups (Aiewsakun and Katzourakis 2016).

We were able to demonstrate that the *Mavericks* of cyprinid fish are under strong purifying selection, with an *ω* of the order of 0.001–0.0001 for all genes. Purifying selection in the order of 10^−3^ has been observed before in dsDNA viruses with the captured CD200 immunoglobulin and CCL3 chemokine genes of rhadinoviruses (family *Herpesviridae*) (Aswad and Katzourakis 2018), but these values seem to be generally low. Since we used branch models for detecting selection, our inference reflects the major evolutionary pressures acting on the whole-gene level. Instances of genes in which the internal/terminal branch model was favored were probably due to the effect of relaxed selection acting on recent pseudogenes, as could be inferred from the higher *ω* for terminal branches. The low *ω* for internal branches for all genes suggests that *Mavericks* have continuously produced viral particles capable of germ-line reinfection throughout their history. Interestingly, the gene *pw* was the only one consistent with a host switch model, indicating that it may be associated with a role in host adaptation since host-switch branches had higher estimated values of *ω*. In the case of human endogenous retroviruses, most elements show evidence of purifying selection on the *env* gene which is consistent with an origin through formation of viral particles and reinfection (Belshaw et al. 2004). On the other hand, the *env* gene of some human endogenous retroviruses evolves neutrally, suggesting the gene is no longer functional in these cases and that these elements have been copied by complementation *in trans* or intragenomic replication *in cis* (Belshaw et al. 2005). Since all the virion morphogenesis homologs are under strong purifying selection in *Mavericks*, this demonstrates they have evolved mainly through formation of viral particles and reinfection, as with the endogenous retroviruses. Overall, we have shown that purifying selection has preserved the functionality of genes from cyprinid *Mavericks* over millions of years.

Inconsistencies between the host and *Maverick* phylogenies within major groups of actinopterygians and tetrapods, indicate that the evolutionary history of *Mavericks* is characterized by a dynamic rather than a stable association with their hosts. Specifically, through our cophylogenetic analyses, we show that these inconsistencies support a history of extensive host switching in cyprinid *Mavericks*. Host switching is a general phenomenon that occurs during viral evolution, and in particular, a strong signal for host switching has been found in the related families of dsDNA viruses *Adenoviridae* and *Poxviridae* (Geoghegan et al. 2017). On the other hand, we have also found evidence for codivergence. Therefore, the evolution of *Mavericks* in vertebrates shows signs of both vertical and horizontal forms of transmission.

The existence of intact elements in the genomes of vertebrates, together with constrained genome sizes and strong evidence for host switching coincident with an intense purifying selection on core genes (and especially those involved in morphogenesis), are robust indications that *Mavericks* are an active lineage of integrative viruses that infect vertebrates. These observations call for incorporation of vertebrate *Mavericks* into the classification of viruses. As group-I and group-II *Mavericks* have been previously shown to be paraphyletic, the proposed term “polintoviruses” (Krupovic et al. 2014) would be taxonomically inadequate. Instead, we propose the creation of the family “Proteoviridae” and the genus “*Alphaproteovirus*” to include vertebrate *Mavericks* and possibly other group-I elements which form a monophyletic group. The family derives its name from the Greek god Proteus, son of Poseidon, who is depicted in the Odyssey as an ancient deity of the sea with the ability to assume the form of different creatures (Homer 1919). The proposed classification of intact elements into 19 species is shown in table 2.

**Table 2.** Proposed Species-Level Classification of Vertebrate *Mavericks* (Family “Proteoviridae,” Genus “*Alphaproteovirus*”), with Designated Type Sequences.

Species	GenBank Acc.	Coordinates
*Anabas testudineus alphaproteovirus*	NW_020535984.1	16773227–16787566
*Anolis carolinensis alphaproteovirus*	NC_014778.1	129158247–129172346
*Astatotilapia calliptera alphaproteovirus*	NC_039306.1	12448581–12465490
*Carassius auratus alphaproteovirus*	NW_020523509.1	38327–56441
*Danio rerio alphaproteovirus 1*	NC_007136.7	27671708–27687993
*Danio rerio alphaproteovirus 2*	NW_001884452.4	163866–182541
*Danio rerio alphaproteovirus 3*	NC_007134.7	16402434–16421359
*Larimichthys crocea alphaproteovirus*	NW_017609269.1	1620911–1634436
*Mastacembelus armatus alphaproteovirus*	OOHQ01000082.1	27018430–27033660
*Oreochromis niloticus alphaproteovirus*	NC_031977.2	37033790–37051003
*Oxygymnocypris stewartii alphaproteovirus 1*	QVTF01001200.1	1425766–1443135
*Oxygymnocypris stewartii alphaproteovirus 2*	QVTF01018600.1	776725–796426
*Perca fluviatilis alphaproteovirus 1*	QFAT01000030.1	1733268–1750442
*Perca fluviatilis alphaproteovirus 2*	QFAT01022094.1	1737–17082
*Pseudocrenilabrinae alphaproteovirus*	NW_020327416.1	45576–61017
*Rhinella marina alphaproteovirus*	ONZH01012121.1	13566–27538
*Terrapene carolina alphaproteovirus*	NW_020664598.1	16371040–16384884
*Xenopus tropicalis alphaproteovirus*	NC_030686.1	6013889–6026611
*Xiphophorus alphaproteovirus*	QPIH01000028.1	6019497–6036410

It is theoretically possible that some sequences derived from proteoviruses may have been exapted to confer advantageous host phenotypes. Co-option of transposable elements is known to have rewired genetic networks and driven changes in the gene expression patterns of vertebrates (Romanish et al. 2007; Kunarso et al. 2010). For example, the insertion of a proteovirus into the promoter region of the growth hormone gene of *Oncorhynchus tshawytscha*, may be in part responsible for the size difference between this species and the smaller *Salmo salar* (von Schalburg et al. 2008). Endogenous proteoviruses might also function as an EVE-derived immunity against their exogenous counterparts, but this remains to be seen since exogenous forms are still unknown.

An intriguing possibility, is that proteoviruses may function as a virophage-derived immunity against NCLDV infections in vertebrates. The nature of antiviral defense systems based on endogenous virophages has been described in detail for unicellular eukaryotes. Fischer and Hackl (2016) discovered that *Mavirus* provirophages in the genome of *Cafeteria roenbergensis* function against infection by *C. roenbergensis Virus* (CroV). CroV infection triggers virophage synthesis, and although this leads to cell lysis, the newly produced virophages inhibit replication of the giant virus in cells of the wider host population (Fischer and Hackl 2016). Therefore, expression of intact endogenous proteoviruses and formation of their virions may depend on activation by a giant virus, thus giving protection at the level of a multicellular organism. The host range of the family *Iridoviridae* is consistent with this hypothesis; iridoviruses are important pathogens of fish, amphibians, and nonavian reptiles (Williams et al. 2005; Chinchar et al. 2017). These are precisely the groups where we identified intact elements, whereas iridoviruses are not known to infect either birds or mammals. Epizootics caused by iridoviruses can reach mortalities of 100% in fish (He et al. 2000) and mass mortality events have been reported for amphibians (Kik et al. 2011). Thus, the extinction of iridoviruses from birds and mammals may have driven the definitive deterioration of proteoviruses in their genomes. On the basis of these dynamics, we hypothesize that the rise of adenoviruses, which attain their greatest diversity in mammals and birds, could have coincided with the elimination of proteoviruses in these groups as well.

We have shown that the *Mavericks* of vertebrates represent an ancient lineage of mostly aquatic dsDNA viruses, which have persisted for hundreds of millions of years by integrating into the genomes of hosts and switching species frequently. The existence of elements with an intact coding capacity together with intense signatures of purifying selection on all genes, strongly indicate that some lineages of vertebrate proteoviruses are functional. Unlike endogenous retroviruses, which are a major component of eukaryotic genomes, it seems proteoviruses are one of the rare instances of endogenous dsDNA viruses in metazoans, such as the *Teratorns*, large elements found in the genomes of teleosts which originate from a *piggyBac*-alloherpesvirus fusion (Aswad and Katzourakis 2017; Inoue et al. 2018). Investigating the ecological roles of proteoviruses remains an important question, especially when they could function as a virophage-based immune system against NCLDVs in a broad range of vertebrates. Molecular characterization of proteoviruses will also be essential, given that the conditions leading to the formation of virions remain unknown. This is particularly relevant since they seem attractive candidates to develop new gene transfer technologies for use in genetic engineering or vaccine development.

## Materials and Methods

### Genome Mining

We selected the genomes of 533 vertebrates available in the NCBI RefSeq and WGS databases (NCBI-Resource-Coordinators 2016) as of December 2018, in addition to the assembly of the Iberian ribbed newt *Pleurodeles waltl* (Elewa et al. 2017), which at the time was not available in the public repositories (this genome sequence was kindly provided to us by A. Elewa). Each genome was screened individually in the NCBI-BLAST server with tBLASTn (Altschul et al. 1990; Johnson et al. 2008) using the integrase (INT) and pPOLB sequences of the *D. rerio* Polinton-1 from RepBase (Bao et al. 2015) as queries; we used local BLAST version 2.8.0+ to screen *P. waltl* (Camacho et al. 2009). A set of candidate regions for downstream analysis was recovered by identifying hits to INT and pPOLB that colocalized to the same contig and were separated by 4–40 kb. The DNA sequences of these regions were downloaded with 20-kb flanks added to each end, giving 3,913 candidates in total.

To map the elements within the candidate regions, we blasted each sequence against itself (BLASTn 2.8.0+) and analyzed the hits producing alignments of inverted repeats, which were identified as alignment pairs with hit-coordinates in reverse orientations. From 2,863 sequences with inverted repeats, 628 had the 5′-(AG)_3_//(CT)_3_-3′ and 5′-(AGT)_2_//(ACT)_2_-3′ end-motifs previously reported for vertebrate *Mavericks* (Kapitonov and Jurka 2006), as well as perfect 6-bp target site duplications (TSDs). These sequences were partitioned according to species/end-motif and aligned using MAFFT version 7.407 (Katoh et al. 2002). We used the alignments to extract the first 200 bp of the 5′ terminal inverted repeat and build nucleotide hidden Markov models (HMMs) with hmmbuild version 3.2.1 (Eddy 1995, 2009). Next, we used the models for each species/end-motif to find additional inverted repeats and TSDs in the remaining candidates within that species using nhmmer version 3.2.1. New elements were aligned to the initial species/end-motif partitions (MAFFT) and refined HMMs were obtained, these were used in turn as heterologous probes to annotate TIRs between species. A final set of alignments and HMMs was then used to screen all the remaining sequences. This strategy allowed us to map the locations of 1,345 *Mavericks* with recognizable TIRs (which include both elements with perfect and imperfect repeats).

### Gene Prediction

Genes in the candidate sequences were modeled using Genewise version 2.4.1 (Birney et al. 2004) and protein HMMs of the eight core genes of vertebrate *Mavericks* (*int*, *ppolb*, *atp*, *pro*, *major cp*, *minor cp*, *pz*, and *pw*). Specifically, we compiled all the predictions from the RepBase consensus sequences (Bao et al. 2015), clustered them using MMseqs2 (Steinegger and Soding 2017), and assigned them to homologous protein sets using HHpred (Soding et al. 2005; Zimmermann et al. 2018). These groups were used to query the nonredundant protein database in BLASTp searches (restricted to “vertebrates” and *e*-value <1e^−100^, this very conservative *e*-value was chosen to ensure that only homologous proteins would be included in the reference alignments used for gene modeling). Results were combined and used to search the nonredundant nucleotide database with the command line tBLASTn. Protein sequences were aligned with MAFFT and HMMs were built in HMMer2 format (hmmbuild version 2.3.2) for compatibility with Genewise.

We removed the host DNA from the *Maverick* candidates using the genomic coordinates of the inverted repeats found in the previous step. These sequences were masked with RepeatMasker version 4.0.8 (Smit et al. 2013–2015) using the Dfam 2.0 (BLAST engine) (Hubley et al. 2016) and RepBase (release 20181026, hmmer engine) repeat libraries with *Mavericks*/*Polintons* removed. Although it was possible to mask secondary transposon insertions in several species, this was not possible for all since they are not represented in the databases. Further, comparisons in RepeatMasker are made at the nucleotide level which would produce conservative maskings.

The protein HMMs were used in Genewise (genewisedb mode) to model the core genes of the masked sequences. In addition, regions which showed positive hits to INT and pPOLB but lacked inverted repeats were also included in gene modeling, as these could represent defective copies with relevance to reconstructing the evolutionary history of *Mavericks*.

### Protein Multiple Sequence Alignments

Protein predictions were extracted from the Genewise output files and if fragmented, concatenated to other sequence fragments belonging to the same prediction. We assembled multi-FASTA files with unique predictions for each homologous protein. All sets proved difficult to align given that many sequences were not masked, some presumably had indel mutations and there were possibly false positive fragments in these predictions (especially short stretches to the N-terminal end, separated by long “introns” to the main prediction). We used six different approaches to choose appropriate alignments for phylogenetic analysis.

Initial sets were aligned using MAFFT, Clustal Omega (two iterations) (Sievers et al. 2011), PASTA (Mirarab et al. 2015), and FAMSA (Deorowicz et al. 2016). Columns with ≥50% gaps in the resulting alignments were removed. We noticed that trimAl (Capella-Gutierrez et al. 2009) was not able to recover conserved positions from either the Clustal Omega or FAMSA alignments, so these were discarded. It was possible to trim both the MAFFT and the PASTA alignments, which we did using the automated1 heuristic; this option chooses between the three modes of automatic parameter selection depending on alignment characteristics (Capella-Gutierrez et al. 2009). The other two approaches consisted of “refinements” of the MAFFT alignments since these were seen to give the best summary statistics (explained below).

First, we aligned sequences with either default MAFFT or MAFFT with the *–*leavegappy flag (which introduces fewer gaps in gap-rich regions). We then removed columns with ≥99% gaps, unaligned the sequences, and realigned them using the previous settings. Next, we removed columns with ≥50% gaps as before. At this stage, a subset of the main alignment with sequences having ≤10% gaps was trimmed in trimAl (automated1). This subset alignment was used as a scaffold to add the removed sequences with the –add and –keeplength functions of MAFFT, thus giving a merged final alignment with all sequences.

As criteria to choose the final alignments, we considered basic statistics from the SQUID package (Eddy 2002): alignment length, mean, maximum and minimum sequence lengths, residue content, average identity, most related/unrelated pair, and most distant sequence. Similarly, we computed the mean Shannon entropy (ignoring gaps and missing symbols) (Shannon 1948) for each alignment as well as the mean observed, Jukes and Cantor, Poisson and Gamma distances in MATLAB (MathWorks, Inc. 2019) for all pairwise comparisons. We chose the alignments that approached the expected protein length, had identifiable conserved motifs reported in the literature (Gao and Voytas 2005; Kapitonov and Jurka 2006; Pritham et al. 2007; Krupovic and Bamford 2008; Zhou et al. 2010; Haapa-Paananen et al. 2014; Krupovic et al. 2014), the highest identities and residue content as well as the lowest entropy and pairwise distances. The best alignment approach for most protein sets was MAFFT followed by trimming, whereas in the case of INT the best approach was MAFFT with realignment and trimming.

### Orthology

To assess orthology of the predictions, we extracted 2 kb of DNA from the flanks of each element. We then used BLASTn to query the database of all flanks and recovered loci which showed hits to both flanks. Candidates were downloaded in full, such that they contained a continuous sequence including both flanks and the elements, and aligned them using the progressive algorithm in Mauve version 2015-02-25 (Darling et al. 2004). In alignments involving elements without TIRs, the limits between host and *Maverick* DNA are not clear, so we walked through these loci by adding 10 kb to each flank and aligning once again in Mauve. The alignments were visualized and only those which showed consistent high similarity as well as colinear blocks were considered to be orthologs.

### cDNA Multiple Sequence Alignments

We used the initial cDNA predictions recovered from Genewise to derive codon alignments for each gene. First, updated HMMs were built for each protein from the final alignments. These models were used to obtain the homologous coding subsequence from the initial cDNA predictions. Stretches of modeled codons were seen to be either contiguous or separated by short <100-bp “introns” (possibly insertions not included in the model). Sequences were pooled and aligned using TranslatorX (Abascal et al. 2010), which aligns coding DNAs by translating them and using the aminoacid alignment as a guide for the codons, such that all the sequences are in the same reading frame. Sequences were edited manually to remove insertions/nonhomologous regions.

### Phylogenetic Analysis

The initial alignments contained thousands of sequences and probably had some redundancy from many sequences that formed well-supported clades within the same host genomes, for these reasons, we implemented an approach to choose a phylogenetically informative subset in order to reduce the computational expense of downstream analyses. We used Fasttree version 2.1.11 (Price et al. 2010) with the JTT+CAT model and Shimodaira–Hasegawa support to approximate a maximum likelihood phylogeny for each protein. We selected subsets of sequences ensuring that protein predictions from all genomes were represented, as well as all the orthologs and elements with apparently intact genes. In particular, we chose subsets from groups that were monophyletic with respect to the same host genome and support values that were >0.70, also making sure that each clade was represented by at least three sequences. In cases such as in salmonids, chelonians, and crocodilians, where the sequences did not separate into well-defined clusters by species (but were nonetheless monophyletic as a whole), we tried to obtain a representative sample from the topology although which specific sequence was selected was rather subjective. We then performed another round of sequence selection using maximum likelihood trees. Before running these analyses, we used ModelTest-NG version 0.1.5 (Darriba 2020) to select the best fitting models of protein evolution. Both the minimum AIC and BIC criteria agreed on the JTT+G model for all proteins, with estimated frequencies (+F) for the ATP, PRO, and pPOLB alignments. Analyses were run on IQtree version 1.6.11 (Nguyen et al. 2015) with the selected models and 1,000 ultrafast bootstrap replicates. From these alignments, a final subset was chosen with the same criteria outlined above.

Finally, we used BEAST2 version 2.5.2 (Bouckaert et al. 2014) to perform Bayesian inference on the tree topology, divergence times, and rates of evolution for each of the final alignments. For each group of *Maverick* orthologs, we calibrated the prior age distribution of their most recent common ancestor with the age of the most recent common ancestor inferred for hosts in the literature. We set the prior mean equal to the reported mean, adjusting the deviation such that the lognormal distribution contained the lower and upper bounds of the 95% confidence interval. The points and references used for all the prior calibrations are provided in the supplementary excel file 2, Supplementary Material online. The best models of protein evolution were selected as before. Analyses were started with the maximum likelihood topology estimated in RaxML-NG (Stamatakis et al. 2005; Kozlov et al. 2019) and run in parallel with logs made every 5,000 generations. As the choice for the molecular clock, we used an uncorrelated relaxed clock (Drummond et al. 2006) using the lognormal distribution, which allows each branch in the tree to have its own evolutionary rate. We did this to relax the rate assumption since *Mavericks* could have complex evolutionary dynamics alternating between endogenous and exogenous modes. Runs were combined in LogCombiner using a 1–25% burn-in and inspected in Tracer (Rambaut et al. 2018) ensuring that estimates had effective sample sizes ∼200 or greater, good mixing, and convergence (MCMC chain lengths were between 345 million and 4,241 million generations). Final tree files were combined by resampling at a lower frequency in LogCombiner and summarized as Maximum Credibility trees in TreeAnnotator. The resulting time trees were visualized in Figtree version 1.4.4 (Rambaut 2018). To confirm the robustness of our results, we computed an independent Bayesian tree in MrBayes 3.2.7a (Huelsenbeck and Ronquist 2001), using the DNA polymerase of intact *Mavericks* exclusively (MCMC length: 2,000,000 generations, model: LG+I+G4).

### Cophylogenetic Analysis

In order to test for possible host switches and the selective regimes affecting vertebrate *Mavericks*, we chose to focus on the elements from cyprinid fish (Cypriniformes: Cyprinidae). The reasons for this are that multiple cyprinids harbor *Mavericks*, some elements have an intact coding capacity, they have attained considerable copy numbers (e.g., in *O. stewartii*) and their phylogeny was well supported but inconsistent with that of their hosts. The tree topology for each gene was analyzed separately and the host tree was built by combining results from the relevant literature (Wang et al. 2012; Yang et al. 2015; Yang et al. 2016). We adopted a simulation-based approach using ABC as implemented in Coala 1.2.1 (Baudet et al. 2015) to quantitatively assess the importance of four classes of cophylogenetic events: cospeciation (simultaneous speciation of parasites and host), duplication (speciation of parasites without host speciation), host switch (parasite jumps from one host species to another), and loss (of a parasite from a host lineage after speciation). Analyses were run for five rounds to attain convergence (without overfitting) and using tolerance thresholds of 0.1 in the ABC rejection algorithm. The mean probability of each event class was then used to calculate associated costs (cost_*i*_ = −log_e_(*p_i_*)), for the computation of the most parsimonious reconciliation tree in Mowgli (Doyon et al. 2010). This reconciled tree was visualized in Sylvx (Chevenet et al. 2016).

### Selection Analysis

As a measure of the selective regimes influencing the evolution of these sequences, we estimated the nonsynonymous to synonymous substitution ratios (*ω*) under the maximum-likelihood framework of the CODEML package from PAML 4 (Yang 2007). We used unrooted subtrees of the protein Bayesian phylogenies for cyprinids as the *Maverick* topologies and the codon-aligned cDNA data as described above. Several models were tested: a single-ratio model with *ω* = 1 (the neutral expectation), a single-ratio model with *ω* = estimated, and a two-ratio model for internal and terminal branches (*ω*_internal_ = estimated, *ω*_terminal_ = estimated). Our alternative hypothesis is that episodes of diversifying selection may have occurred coinciding with host switches, thus, we used the tree reconciliation computed in Jane (Conow et al. 2010) to label the branches involved in host switches in a two-ratio model (*ω*_switch_ = estimated, *ω*_no-switch_ = estimated). A likelihood-ratio test was conducted for each pair of nested models, and the χ^2^ test statistic compared with its critical value (under the difference of the degrees of freedom) to assess the significance of these comparisons. Nonnested models were compared using the Akaike information criterion.

## Supplementary Material

Supplementary data are available at *Molecular Biology and Evolution* online.

## Supplementary Material

msaa291_Supplementary_Data
